# Understanding the Slow Depletion of Memory CD4^+^ T Cells in HIV Infection

**DOI:** 10.1371/journal.pmed.0040177

**Published:** 2007-05-22

**Authors:** Andrew Yates, Jaroslav Stark, Nigel Klein, Rustom Antia, Robin Callard

**Affiliations:** 1 Department of Biology, Emory University, Atlanta, Georgia, United States of America; 2 Centre for Mathematics and Physics in the Life Sciences and Experimental Biology, University College London, London, United Kingdom; 3 Department of Mathematics, Imperial College, London, United Kingdom; 4 Centre for Integrative Systems Biology at Imperial College, Imperial College London, United Kingdom; 5 Immunobiology Unit, Institute of Child Health, London, United Kingdom; Utrecht University, The Netherlands

## Abstract

**Background:**

The asymptomatic phase of HIV infection is characterised by a slow decline of peripheral blood CD4^+^ T cells. Why this decline is slow is not understood. One potential explanation is that the low average rate of homeostatic proliferation or immune activation dictates the pace of a “runaway” decline of memory CD4^+^ T cells, in which activation drives infection, higher viral loads, more recruitment of cells into an activated state, and further infection events. We explore this hypothesis using mathematical models.

**Methods and Findings:**

Using simple mathematical models of the dynamics of T cell homeostasis and proliferation, we find that this mechanism fails to explain the time scale of CD4^+^ memory T cell loss. Instead it predicts the rapid attainment of a stable set point, so other mechanisms must be invoked to explain the slow decline in CD4^+^ cells.

**Conclusions:**

A runaway cycle in which elevated CD4^+^ T cell activation and proliferation drive HIV production and vice versa cannot explain the pace of depletion during chronic HIV infection. We summarize some alternative mechanisms by which the CD4^+^ memory T cell homeostatic set point might slowly diminish. While none are mutually exclusive, the phenomenon of viral rebound, in which interruption of antiretroviral therapy causes a rapid return to pretreatment viral load and T cell counts, supports the model of virus adaptation as a major force driving depletion.

## Abstract 2

Using a simple mathematical model, Andrew Yates and colleagues show that a runaway cycle of T cell activation and infection cannot explain the slow rate of CD4 decline during chronic HIV infection.

## Editors' Summary

**Background.:**

The AIDS virus causes disease by inactivating the body's immune responses. Most severely affected are the white blood cells known as T lymphocytes, particularly the CD4^+^ T cells that recognize infection and enable other cells of the immune system to respond against it. Following rapid loss in the first few weeks of HIV infection, the number of CD4^+^ cells in the blood falls slowly, often over a period of ten years or longer. The exact mechanisms by which CD4^+^ cell depletion occurs are not fully understood.

**Why Was This Study Done?:**

For over a decade, researchers have used a “tap and drain” analogy to describe CD4^+^ cell loss. In this description, CD4^+^ cells (like water in a sink) are constantly being eliminated by HIV (the drain), while the body is constantly replacing them with new ones (the tap). Over time, the tap cannot keep up with the drain, and CD4 counts begin to drop, leaving the body susceptible to the infections that define AIDS.

CD4^+^ cells that are activated in response to invading microbes (such as HIV) are highly susceptible to infection with HIV, and following infection these cells may produce many new copies of HIV before dying. Therefore, one popular explanation for CD4^+^ cell loss is the “runaway” hypothesis, in which CD4^+^ cells infected by HIV produce more virus particles, which activate more CD4^+^ cells that in turn become infected, leading to an ongoing cycle of CD4^+^ cell activation, infection, HIV production, and cell destruction. The authors wanted to investigate whether this hypothesis can explain why CD4^+^ cell levels fall slowly.

**What Did the Researchers Do and Find?:**

Using a mathematical model (a series of equations to describe the processes by which CD4^+^ cells are produced and eliminated), the authors show that, if the “runaway” hypothesis were correct, then CD4^+^ cells would fall to low levels over a few months, not over several years. They therefore conclude that the “runaway” hypothesis cannot explain the slow pace of CD4^+^ cell depletion in HIV infection.

**What Do These Findings Mean?:**

These results show that a slow process must be active in the HIV-induced depletion of CD4^+^ cells. Because of its simplicity, the model used in this study is convincing in its rejection of the runaway hypothesis, but a more detailed analysis will be needed to tell us precisely what the slow process is. One possibility raised by the authors is that this process is the slow adaptation of the virus itself over the course of infection. Specifically identifying this process will provide a key insight into the nature of HIV disease and indicate potential new approaches to therapy.

**Additional Information.:**

Please access these Web sites via the online version of this summary at http://dx.doi.org/10.1371/journal.pmed.0040177.

- Read the related Perspective on this article by Rob J. de Boer
- University of California, San Francisco's HIV InSite Knowledge Base includes information on HIV immunology
- The National Institute of Allergy and Infectious Diseases provides AIDS fact sheets and brochures that cover a variety of topics

## Introduction

Untreated HIV infection in humans is characterised by extensive depletion of CD4^+^ T cell subsets. In the acute phase, virus appears to preferentially and efficiently target HIV-specific [1,2] and effector-memory CD4^+^ T cells that reside in mucosal tissue [3]. Studies of macaques infected with SIV show the massive loss of memory-phenotype CD4^+^ cells in the gut [4], which comprise most of total body CD4^+^ cells. Studies in HIV-infected humans mirror these observations [5–7]. Following this huge systemic insult, the untreated disease enters a chronic phase in which what remains of the CD4^+^ T cell compartments is slowly eroded over a time scale of several years. What causes this slow loss is not known.

There is considerable loss of uninfected cells in the chronic phase [8–12], suggesting that the decline is unlikely to be due solely to the direct cytopathic effects of the virus [13] or the removal of infected cells by cytotoxic T lymphocytes (CTLs) [14]. Chronic immune activation (IA) is frequently cited as an erosive force [15–18], although it remains more of a strong association than a detailed mechanism. Elevated levels of polyclonal CD4^+^ T cell activation and proliferation markers are observed in blood [19–22], and levels of T cell activation are the strongest predictor of the rate of progression to AIDS [23].

This increase in IA and immunopathology may be a direct result of the immune cell interactions with HIV. An alternative hypothesis is that it is a secondary effect—the acute mucosal lymphopaenia causes the relocation of immunogenic gut antigens and systemic responses [24]. In either case, IA may have diverse effects on both division and death rates of T cells of all specificities; altering cytokine environments and circulation patterns, driving cells into division and towards senescence through repeated stimulation, or rendering them susceptible to activation-induced cell death. Further, particularly given the extent of the initial depletion, some degree of homeostatic compensation will also contribute to increased average rates of division in CD4^+^ T cell compartments.

Nevertheless, the causal link between the increased CD4^+^ turnover and the slow decline in the blood remains unclear. The average rates of division and death (turnover) of all CD4^+^ subpopulations are increased, and these rates are fast compared to the overall rate of T cell depletion. These two key observations are often restated in terms of a “tap and drain” analogy [25] in which a small imbalance between the net rates of replenishment and death drives an inexorable decline to a state of low CD4^+^ T cell numbers. Yet we still need to explain the mechanism underlying this imbalance, or the failure of T cell homeostasis.

The increased susceptibility of activated or proliferating T cells to infection [26] suggests a specific and plausible candidate mechanism. Both IA (a response to antigenic stimulation) and homeostatic proliferation (a response to lymphopaenia) may fuel the fire by generating new susceptible cells and thus more infection. Elevated turnover results in more infection, possibly more IA or homeostatic compensation, and runaway depletion of CD4^+^ T cells. Specifically, can this mechanism provide the slow time scale for decline of peripheral blood CD4^+^ memory T cells? In this paper we use a simple mathematical model of T cell dynamics to address this question. We begin by constructing a model of memory CD4^+^ T cell maintenance in health, and discuss how it changes in the presence of HIV.

## Results

### Homeostasis of Memory CD4^+^ T Cells in Health

The CD4^+^ T cell pool comprises naive (antigen-inexperienced) cells and heterogeneous memory (antigen-experienced) populations. The mechanisms by which memory cells are maintained are not completely defined [27], but survival and self-renewing proliferation are likely to be controlled by competition for homeostatic resources such as cytokines and TCR-self-peptide/MHC interactions, and rates of turnover may vary between memory subpopulations [28]. In uninfected individuals, depletion of CD4^+^ T cells results in compensatory homeostatic proliferation [29]. This observation has led to the concept of T cell “niches,” either at the level of the memory cell compartment as a whole [30], or at the level of individual clones [31].

**Figure 1:**
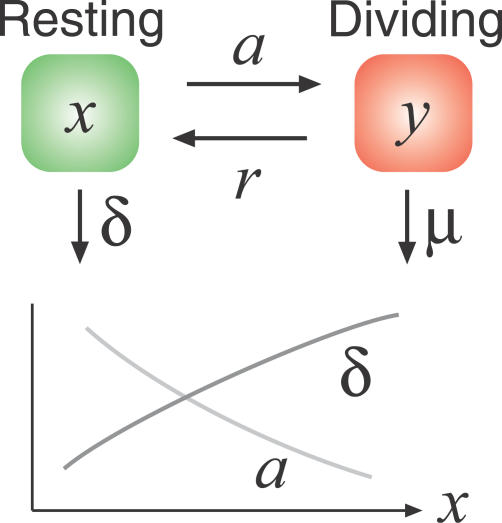
A Simple Model of Self-Renewing Memory CD4^+^ T Cell Homeostasis in the Absence of HIV Infection, with Density-Dependent Rates of Division and Death of Resting Cells 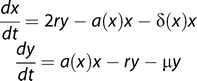

With HIV infection in mind, and building on earlier studies [32,33], we propose a model of healthy memory CD4^+^ T cell homeostasis that distinguishes between resting cells and cells that have received homeostatic division signals and so are transiently activated (Figure 1).

We assume that resting memory CD4^+^ T cells (*x*) compete for homeostatic division signals and receive them at an average rate *a*. The mean interdivision time of memory CD4^+^ T cells in healthy humans (1/*a*) has been estimated using deuterated glucose labelling and is approximately 15 d for effector memory CD4^+^ T cells, 48 d for central memory cells [34], or 26 d when averaged over the two compartments [35]. Earlier estimates by other methods suggest it may be as high as 150 d [36]. We represent competition by assuming that this time scale decreases (in other words, *a* increases) under lymphopaenic conditions, as observed experimentally in mice (unpublished data). We assume that the death rate of resting memory CD4^+^ T cells (*δ*) is low [36,37] and density dependent, falling further under lymphopaenic conditions as survival signals become more abundant. Our conclusions are weakly dependent on the density dependence of *a*(*x*) and *δ*(*x*). For the simulations we show below, we used the linear forms *a*(*x*) = *a*(1 − *x*/*κ*) and *δ*(*x*) = *δ*_0_ (*x*/*κ*)*,* where *κ* is a constant that scales the size of the memory T cell population. With our choice of parameters, in a “full” memory CD4^+^ compartment the mean time between divisions is 60 d, decreasing to 30 d under lymphopaenic conditions, and the average lifetime of resting cells is 400 d.

In our model, cells that have received a homeostatic division signal enter an actively dividing state (*y*) and then return to the resting state at rate *r,* dividing once as they do so. The inverse of *r* is the average time for transit through the cell cycle from a resting state, which we choose to be 24 h, or *r* = 1 d^−1^ [38,39]. Again, our results are not sensitive to this choice. To maintain homeostasis, our assumption of a low death rate of resting cells implies that homeostatically dividing cells are much more susceptible to death, as has been confirmed in studies of T cell turnover [35]. Using activation markers as an indicator of progression through the cell cycle, 0.5%–2% of CD4^+^ memory cells are in an activated or dividing state *y* at any time in spleen and blood [28,40], which we use to constrain the death rate of activated cells (*μ*) given *a* and *r*.

Our key observation here is that the rate *a*(*x*) determines the time scale for homeostatic turnover or recovery from lymphopaenia. Even if we conservatively use the slowest estimates of memory turnover rates and we neglect the increase in homeostatic proliferation observed under lymphopaenia, our basic conclusion is unchanged. We describe it in the following section.

### HIV and Memory CD4^+^ T Cells—The “Slow Runaway” Hypothesis

**Figure 2:**
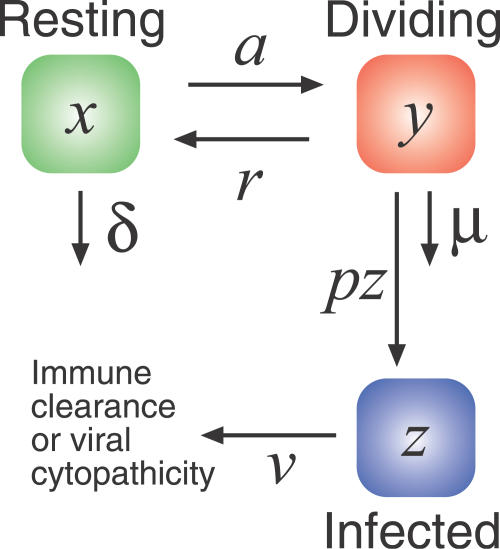
Extending the Previous Model of Memory CD4^+^ Homeostasis to Include HIV Infection We now assume that activated cells are infected at a rate proportional to the virus load (which in turn is assumed to be proportional to the productively infected cell count *z*) and an infectivity parameter *p* that models the efficiency of the infection process. 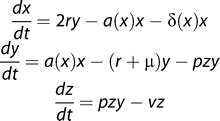

We now propose a minimal model of the interaction between HIV and memory CD4^+^ homeostasis (Figure 2).

We assume that homeostatically activated or dividing cells (*y*) are more susceptible to HIV infection than resting cells (*x*). We also assume that infection of the homeostatically dividing cells occurs at a rate proportional to both the average viral infectivity *p,* a parameter that represents the efficiency of infection, and the viral load, which we take to be proportional to the number of infected cells at any time *(z)*. Infected cells have a short lifetime (*v* ≈ 0.5 d^−1^ [41]). For now we assume resting cells cannot be infected, but this assumption can be relaxed without affecting our conclusions.

**Figure 3:**
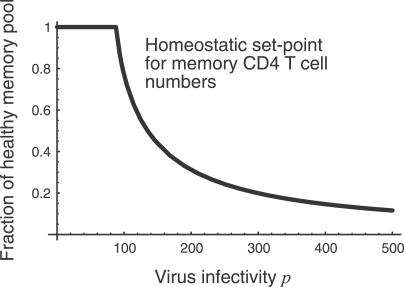
Steady-State Pool Size as a Function of Virus Infectivity *p* We choose units of peripheral blood cell counts such that healthy memory CD4^+^ T cell numbers are unity. Below a threshold value of *p* the infection fails to take hold. We expect HIV infections to correspond to the area right of this threshold.

**Figure 4:**
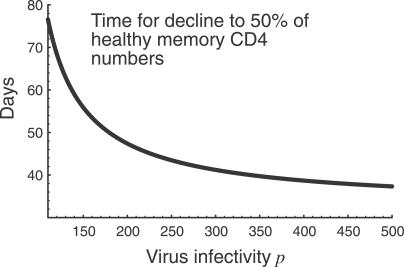
The Predicted Time for Memory CD4^+^ T Cell Numbers to Decline from 100% to 50% of Healthy Numbers, as a Function of Virus Infectivity *p* Parameter values (see model equations in caption to Figure 2): *a*(*x*) = *a*_0_(1 − *x*/*κ*), where *a*_0_ = 1/30 d^−1^, giving *a*(x*) ≈ 1/60 (here x* refers to the steady state numbers of resting memory CD4^+^ T cells in an uninfected individual); *κ* = 1; *δ*(*x*) = *δ*_0_ *x*/*κ,* where *δ*_0_ = 1/200, giving *δ*(x*) ≈ 1/400; *μ* = 1.06; *v* = 0.5; and *r* = 1 (all rates in units of d^−1^).

We consider the dynamics of the T cell pool from the beginning of the chronic phase of infection. Our model predicts that total memory CD4^+^ T cell numbers (*x* + *y* + *z*) decline to a set-point which is inversely related to *p* (Figure 3) and increases with *v*. Moreover, the approach to this set point is rapid, on a time scale of weeks to months, a time scale determined given by the average time between homeostatic divisions, 1/*a* (Figure 4). In other words, homeostatic activation-driven infection alone cannot explain the observed rate of memory CD4^+^ decline in HIV infection. Adding other factors such as infection of resting cells increases the rate of approach to the steady state. We note that even the slowest published estimates of CD4^+^ memory T cell turnover rates are too rapid to explain the slow progression of HIV infection as a transient decline to a state of low T cell numbers.

### The Effect of Immune Activation

This model does not include immune-activated cells, which may be recruited from naive or memory CD4^+^ populations. Immune-activated (which we define as either antigen- or bystander-activated) cells make the dominant contribution to increased T cell turnover during HIV infection, which decreases rapidly and substantially when viral replication and new infection is blocked by ART [19]. This reduction in IA and turnover presumably leaves homeostatic compensation as the dominant source of elevated turnover during treatment.

**Figure 5:**
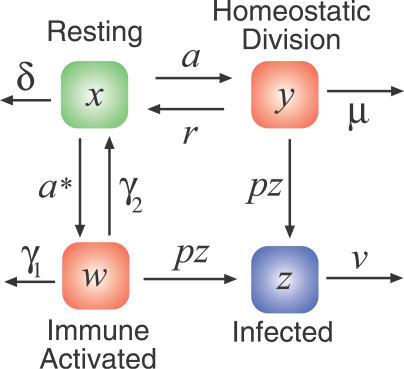
Extending the Model of Memory CD4^+^ T Cell Dynamics in HIV Infection to Include Both Homeostatically Activated (*y*) and Antigen- or Bystander-Activated Cells (*w*) Resting memory cells can undergo homeostatic proliferation at rate *a,* as before; they can also be antigen- or bystander-activated at rate a* and undergo a fold expansion *f* in the process. These cells are infected at rate *pz,* die at rate *γ*_1_, or return to a resting memory state at rate γ_2_. The results we present here are insensitive to the values of *γ*_1_, *γ*_2_, and *f,* which we take to be 0.2 d^−1^, 0.02 d^−1^, and 100, respectively. 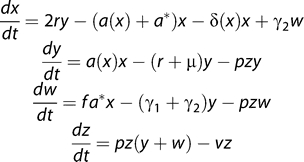

Chronic T cell activation comprises several processes—recruitment from naive or resting memory CD4^+^ compartments, rapid proliferation, and death, either through activation-induced cell death or by cytopathic infection. Immune-activated cells may also return to a resting memory state. In other words, we argue that the chronic IA picture is essentially of a form similar to Figure 2, with the addition of more extensive division and death of activated (effector-phenotype) cells. To explore its effect on the dynamics of the T cell pool as a whole, we extended the model to include IA as well as homeostatic proliferation (Figure 5).

The average rate of antigen or bystander activation (a*) per memory CD4^+^ T cell in untreated HIV infection is not known. The death rate *γ*_1_ has been estimated for acute LCMV infections [37] and is of the order 0.2 d^−1^ for antigen-specific CD4^+^ T cells. This estimate may be reduced under conditions of chronic stimulation as in HIV infection. We choose the rate of return to resting memory *γ*_2_ to be 10% of the effector cell death rate. However, our results are insensitive to the choice of *γ*_1_ and *γ*_2_.

**Figure 6:**
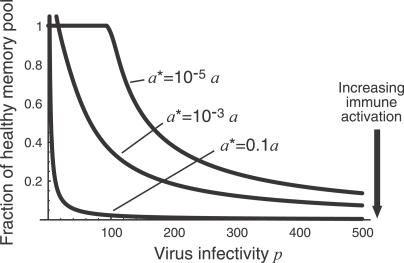
The Predicted Steady-State Pool Size as a Function of Virus Infectivity *p,* in the Presence of Different Levels of Immune Activation a*

**Figure 7:**
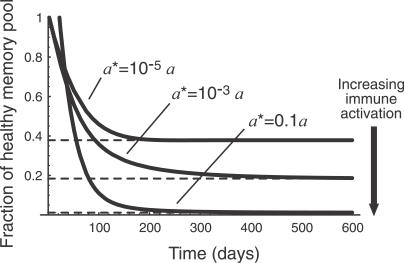
The Predicted Time Course of CD4^+^ Memory T Cell Numbers as They Decline to Their Steady State Level for Different Levels of Immune Activation a* in the Presence of HIV Steady state levels are indicated by dashed lines. The infectivity parameter *p* = 200; other parameters are as in previous figures.

When a* is comparable to or larger than the rate of homeostatic proliferation *a,* the approach to a dynamic equilibrium is even more rapid than in the simpler model (and the set point is lower for a given set of parameters *p* and *v*) as might be expected intuitively (simulation results not shown). In Figures 6 and 7 we illustrate the predictions of the model when the average rate of IA, a*, is smaller than the homeostatic activation rate *a*. In this case we see that the approach to a steady state is still too rapid to explain the progression of chronic HIV infection. The key point here is that in the presence of low average rates of IA, homeostatic activation provides a lower bound on the average time between T cell activation events and so determines the time scale of equilibration of the memory CD4^+^ T cell compartment. Thus other mechanisms must be invoked to explain the slow failure of homeostasis in HIV infection.

### Latent Infection

Resting cells may be infected—potentially with lower susceptibility—and generate virus upon activation. Including latent infection and assuming that these cells behave as normal resting memory cells, our conclusions are unchanged. Latently infected cells may be required to account for viral “rebound” after cessation of ART (see Discussion).

### Thymic Output and Naive Cells

The results we describe above are robust to adding small influx terms into either resting or activated memory CD4^+^ compartments from the naive population. Naive CD4^+^ T cells show very little homeostatic turnover under normal conditions and are likely to be replenished largely by thymic output, which may be significantly impaired in HIV infection [42]. Further, under lymphopaenic conditions, naive T cells may proliferate and acquire an activated or memory-type phenotype [43]. The role of thymic output as a factor controlling the rate of disease progression has been discussed previously [44]. However, in our model the rapid rate of approach to an equilibrium memory pool size is not significantly perturbed by small rates of influx from other compartments.

### Model Predictions

The predicted dependence of steady state pool size with *p* allows comparisons to be drawn with other measurements of virus infectivity in the literature. In our model the key quantity is the rate of infection per activated CD4^+^ T cell, or *pz,* where *z* is the number or density of productively infected cells. In models used extensively elsewhere in the HIV literature (see, for example, [45]), the corresponding quantity is usually denoted *kV,* where *V* is the concentration of virus in plasma and *k* is a “force of infection.” Müller et al. [46] estimate *kV* to be in the range 0.01–0.5 d^−1^ from in vivo data (actually the quantity 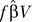 in their notation). Dixit and Perelson [47] estimate rates of infection in vitro and quote comparable values of *kV* ≈ 0.02 − 1 d^−1^, although these estimates should probably be increased for populations of activated target cells. Our model predicts the equivalent quantity (*pz*) to be in the range 0–0.2 d^−1^ for 100 < *p* < 1,000 and for all levels of IA. Steady state numbers of cells at around 50% of healthy numbers correspond to values of *pz* of approximately 0.1 d^−1^, in reasonable agreement with these studies.

Qualitatively, we note that our model predicts that even after inhibition of viral replication with ART (that is, reduction of *p*), average death rates will remain higher than healthy levels during the return to a higher homeostatic set point. This prediction of elevated cell death even during the recovery of cell numbers arises because in our model proliferating cells are more susceptible to death than are resting cells, as has been noted previously in other HIV modelling studies [48,49].

## Discussion

We have used a simple and robust model to reject a mechanism for CD4^+^ T cell decline in chronic HIV infection. However, the model has two key limitations. First, while it distinguishes between quiescent and proliferating memory CD4^+^ T cells, it neglects other potential sources of phenotypic heterogeneity within this population with respect to activation, proliferation, and death. Second, it assumes that cells' replicative capacity remains intact after repeated rounds of stimulation. Chronically stimulated cells may become senescent and progressively lose their ability to proliferate or survive. With these caveats in mind, we can still use the model to frame alternative hypotheses for the mechanism driving the set point in the chronic phase of infection slowly towards an AIDS state. Many hypotheses have been discussed in the literature, all of which involve proposing an additional slow process in T cell population dynamics. We summarise them below in the context of T cell homeostasis.

### 

#### Exhaustion of CD4^+^ subpopulations.

Chronic stimulation may drive the accumulation of a heterogeneous population of T cells exhibiting a nonresponsive or exhausted phenotype, with higher susceptibility to apoptosis and reduced proliferative capacity [50]. This phenomenon is observed among responding T cells in other persistent viral infections, but may also occur among nonspecific (bystander-activated) CD4^+^ T cell populations in HIV infection. In Text S1 we show how an extension to the model of a low constant rate of differentiation of stimulated cells into an exhausted state can generate a slow decline in total T cell numbers.

#### Virus evolution.

Increasing the efficiency of infection, *p,* lowers the memory CD4^+^ set point in our model (Figures 3 and 6), suggesting a mechanism of decline in which progression is driven by slow adaptation of the virus, as has been proposed previously [51]. The infectivity *p* is a compound parameter representing at least two distinct quantities: (i) the probability of productive infection upon encounter between a virion and a susceptible T cell, and (ii) the dependence of plasma virion density on infected cell numbers. The first may change over the course of an infection through a switch in coreceptor usage [52,53] or perhaps changes in receptor expression of chronically stimulated populations. The second may be influenced by virus adaptation (e.g., increases in the numbers of virions produced per infected cell) or the rate of clearance of free virions by antibodies, which may fall as new mutants emerge and/or the availability of T cell help wanes. These processes could be distinguished by assessing whether virus in patients in the chronic phase of infection shows an increasing capacity to infect host CD4^+^ T cells ex vivo over time. We note that this measure of fitness is not necessarily related to virus diversity. Clinical evidence is conflicting in this regard, and the association between diversity and progression is unclear [54–56]. A further possibility is that within-host virus evolution causes a progressive increase in the per capita rate of immune or bystander activation. As can be seen in Figure 7, our model predicts that increasing this rate will lower the steady-state number of memory CD4^+^ T cells.

#### Failure of immune surveillance.

Impaired T cell help and/or CTL exhaustion may decrease the rate of recognition and killing of infected cells by CTLs (contained in the rate constant *v* in the model above) over time [57]. Thus despite the low frequency of infected cells, changes in the efficiency with which they are removed can produce an impact on the T cell compartment as a whole. CTL killing can now be quantified directly in vivo [58] and longitudinal measurements of killing rates may help us to assess whether declining CTL function drives progression. However, this potential mechanism shifts the problem to explaining the slow rate of CTL exhaustion in HIV infection, which is faster in other chronic infections [59].

#### Attrition of homeostatic resources.

Homeostasis may be impaired in HIV infection due to damage to lymphoid architecture [3,60]. Thus chronic inflammation may progressively lower the homeostatic set point by diminishing the availability of T cell proliferation or survival signals. Our model predicts that in the absence of HIV infection, CD4^+^ memory numbers increase with the activation rate *a*—however, they fall with increasing *a* and a* in the presence of HIV, as activation renders more cells susceptible to infection and destruction. This prediction implies that if destruction of lymphatic infrastructure is the source of memory CD4^+^ T cell decline in HIV, its dominant effect on T cell dynamics must be to increase the death rate of activated cells *μ*. The dynamics of activated cells strongly dictate the dynamics of the pool as a whole; the model predicts that increases in the average death rate of activated cells *μ* by as little as a factor of two above healthy levels can lead rapidly to profound depletion in the steady state memory T cell numbers.

#### Mutational meltdown or senescence.

In this model, increased turnover of T cells results in the accumulation of deleterious mutations and impairs their regenerative or survival capacity [61]. The rate at which these mutations accumulate could then determine the time scale of decline. Phenotypic changes induced by repetitive stimulation (e.g., an approach to a terminally differentiated state) may provide a similar mechanism of impairing the average regenerative capacity of the population as a whole. Following a similar argument to that above, a decline in memory CD4^+^ numbers is most likely to be driven by an increase in death rates among persistently stimulated, senescent cells, rather than a decrease in their proliferation rate.

#### Immune hyperactivation.

As we have shown, IA in the presence of HIV can cause a reduction in the set point memory CD4^+^ cell numbers, but the set point for a given level of IA is attained too rapidly to explain the slow depletion of these cells. However, our model predicts that a slow, progressive increase in the per capita rate of activation could cause a downward drift of the set point. Alternatively, and perhaps more plausibly, the immune hyperactivation and heightened homeostatic proliferation may drive a slow increase in the average death rate in the memory population through senescence or exhaustion, as described above [50,61].

## Summary

All of these models seek to explain the imbalance between the average rates of division and death across the CD4^+^ memory pool, and are therefore all variants of the “tap and drain” hypothesis. Distinguishing between them is obviously important for treatment of infection, and while none are mutually exclusive each may contribute to different extents in infected individuals. Additional information, however, comes from the phenomenon of viral rebound—after cessation of ART, the rapid increase of viral load and decline of CD4^+^ counts in peripheral blood to approximately pretreatment levels [62]. Assume that treatment drives the infectivity parameter *p* from an initial value *p*_0_ to a value close to zero, and markedly reduces levels of IA (a* in the models above), which is likely to be an increasing function of the viral load and/or *p*. If reservoirs of latently infected cells persist during treatment, retaining a representative memory of the pretreatment virus population, the virus adaptation model predicts that allowing viral replication to restart will drive the memory CD4^+^ numbers quickly—on a time scale of weeks—to the pretreatment set point determined by *p*_0_, a*, and the remaining (constant) homeostatic parameters. Other models predict the rapid return to the previous set point only if the homeostatic parameters remain constant during treatment—that is, if there is not significant recovery of CTL function or repair of lymphoid architecture or T cell regenerative capacity while virus replication is suppressed.

Clearly the virus adaptation model is not the whole story. Assuming treatment effectively abrogates the production of virus, this model predicts the full reconstitution of the T cell memory compartment during sustained treatment, which is not usually observed. This discrepancy between the virus adaptation model and the observations implies an additional role for lasting impairment to homeostatic mechanisms in HIV infection, perhaps from attrition of homeostatic resources or through differentiation (senescence or exhaustion).

In this paper we have used a model of self-renewing memory CD4^+^ T cell pool to reject the idea of slow runaway decline in HIV infection, in which homeostatic compensation and/or IA drives infection, higher viral loads, more recruitment of cells into an activated state, and further infection events—and intuitively should lead to erosion of the memory CD4^+^ compartment. We argue that this mechanism fails to explain the slow decline of this cell population in the chronic phase of HIV infection. The model indicates that the memory CD4^+^ T cell pool will reach a dynamic equilibrium or set point within weeks or months of the end of the acute infection, a time scale that is dictated by the average time between homeostatic proliferation events. However, the “runaway” model may well be an appropriate description of depletion in the early stages of infection, and particularly among responding HIV-specific cells. Our study highlights how understanding memory CD4^+^ T cell dynamics in chronic HIV infection requires a quantitative description of T cell homeostasis in health, as well as knowledge of how HIV affects the turnover and differentiation of T cell subsets.

## Methods

The ordinary differential equation models used in this study were simulated and analysed using Mathematica [63].

## Supporting Information

Text S1 A Model for CD4 Decline Driven by the Slow Accumulation of Chronically Stimulated, Nonresponsive Cells We illustrate with an extension to the model how adding heterogeneity to the memory CD4^+^ T cell population, in the form of slow differentiation into an exhausted or less responsive phenotype induced by chronic exposure to HIV antigens or inflammatory signals, can induce a slow decline in total cell numbers.
(303 KB PDF)

## Abbreviations

ART: antiretroviral therapy
CTL: cytotoxic T lymphocyte
IA: immune activation
