# Correction: Understanding the Slow Depletion of Memory CD4^+^ T Cells in HIV Infection

**DOI:** 10.1371/journal.pmed.0050011

**Published:** 2008-01-29

**Authors:** Andrew Yates, Jaroslav Stark, Nigel Klein, Rustom Antia, Robin Callard

Correction for:

Yates A, Stark J, Klein N, Antia R, Callard R (2007) Understanding the slow depletion of memory CD4^+^ T cells in HIV infection. PLoS Med 4(5): e177. doi:10.1371/journal.pmed.0040177

In the published manuscript, Figures 3, 4, 6, and 7 were generated using slightly different parameter values than those quoted in the captions. Corrected versions of these figures are shown here. None of the conclusions of the study change as a result.

**Figure 3:**
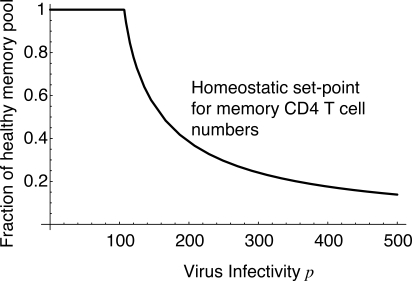
Steady-State Pool Size as a Function of Virus Infectivity *p*

**Figure 4:**
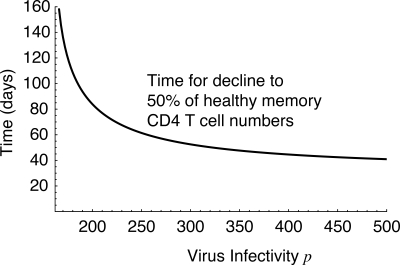
The Predicted Time for Memory CD4^+^ T Cell Numbers to Decline from 100% to 50% of Healthy Numbers, as a Function of Virus Infectivity *p*

**Figure 6:**
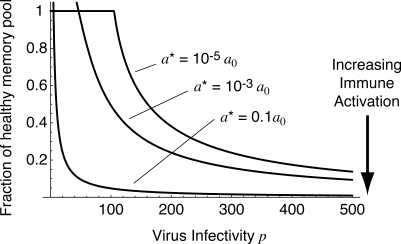
The Predicted Steady-State Pool Size as a Function of Virus Infectivity p, in the Presence of Different Levels of Immune Activation a*

**Figure 7:**
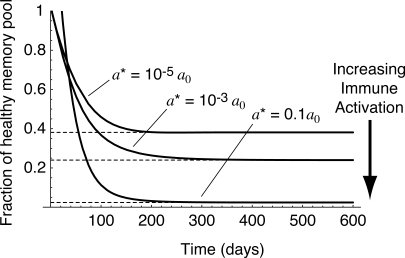
The Predicted Time Course of CD4^+^ Memory T Cell Numbers as They Decline to Their Steady State Level for Different Levels of Immune Activation a* in the Presence of HIV

Other minor corrections:

The homeostatically dividing cell death rate μ is 0.77/day, not 1.06 as stated in the original caption to Figure 4. This rate is calculated using the constraint that approximately 1% of CD4^+^ T cells in blood are activated at the healthy set point, giving


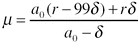


In the third paragraph of the Results section, in the sentence beginning, “For the simulations we show below, we used the linear forms…”, the equation should read *a*(*x*) =*a*_0_ (1−*x*/κ).

At the end of the next paragraph, “given *a* and *r*” should read “given *a*(*x*) and *r*”.

In the caption of Figure 5, the third equation should read *dw*/*dt* = f a^*^ x–(γ_1_ – γ_2_)*w –pzw*.

The authors are exceptionally grateful to Ben Bolker, Jess Beasley, and Carol Chaffee for bringing attention to these errors.

