# Supplementary material for: Understanding the Slow Depletion of Memory CD4+ T Cells in HIV Infection
Source: PLoS Med. 2007 May 22;4(5):e177. doi: 10.1371/journal.pmed.0040177 (PMC1872038; doi:10.1371/journal.pmed.0040177)
Supplement: Supplementary file 1 [file pmed.0040177.sd001.pdf]

Supporting Information S1

We present a simple extension to the model to allow the induction of a non-responsive memory CD4$^+$ T cell phenotype in the presence of HIV infection (Figure S1). This extension requires several additional parameters which cannot be constrained from the literature, but we include it to illustrate a potential mechanism and to show that a decline can be driven by perturbing healthy homeostasis with a slow process. We assume that in the presence of HIV there is a small probability that any given activation event will lead to the irreversible acquisition of an 'exhausted' phenotype with reduced survival time and lower activation rate. The simulation shows how this model can predict the slow accumulation of this phenotype, progressively lowering T cell counts over a timescale of years.

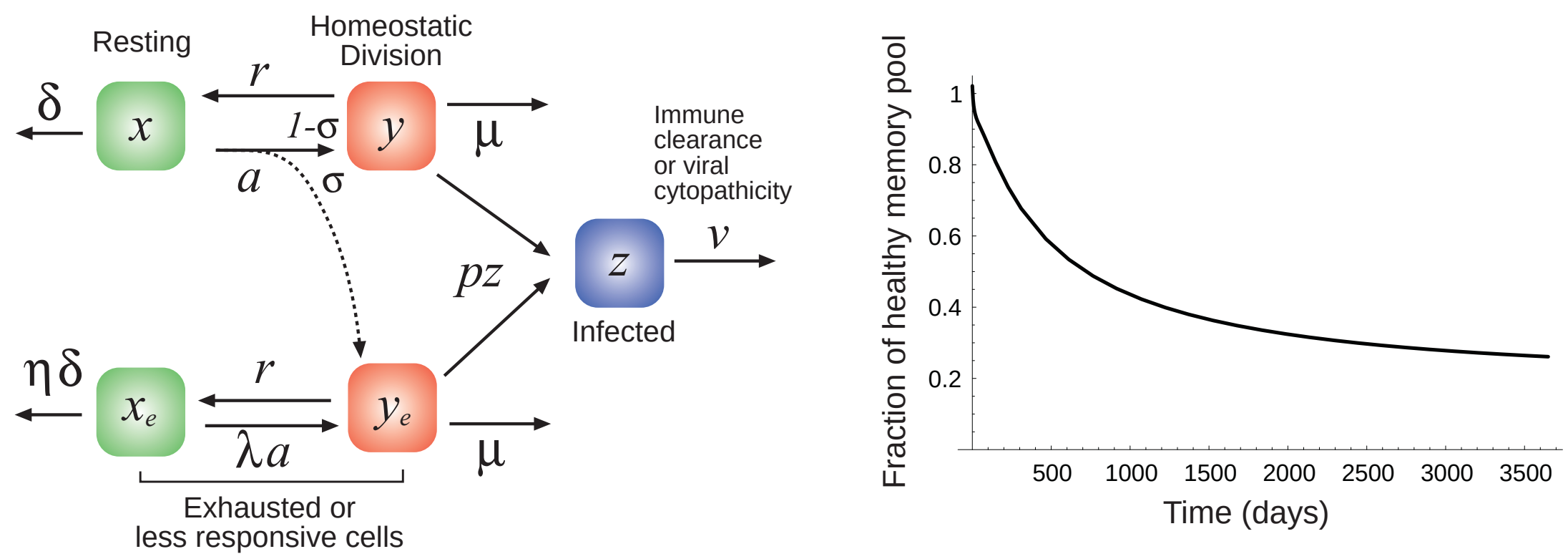

$$
\begin{aligned}
\mathrm{d}x/\mathrm{d}t &= 2ry - a(X)x - \delta(X)x \\
\mathrm{d}y/\mathrm{d}t &= (1-\sigma)a(X)x - (r+\mu)y - pzy \\
\mathrm{d}x_e/\mathrm{d}t &= 2ry_e - \lambda a(X)x_e - \eta\delta(X)x_e \\
\mathrm{d}y_e/\mathrm{d}t &= \sigma a(X)x + \lambda a(X)x_e - (r+\mu)y_e - pzy_e \\
\mathrm{d}z/\mathrm{d}t &= pz(y+y_e) - vz \\
X(t) &= x(t) + x_e(t)
\end{aligned}
$$

Figure S1: Generating a slow timescale of decline of memory CD4$^+$ T cell dynamics in HIV infection by assuming a proportion σ of activated memory T cells enter a non-responsive or exhausted state (variables denoted by subscript e) which have their homeostatic proliferation rate reduced by a factor $\lambda < 1$ and their death rate increased by a factor $\eta > 1$. The parameters used in the simulation above were as Figure 4, along with $\sigma = 0.1$, $\lambda = 0.02$, $\eta = 10$.
